# Glucose metabolism reprogramming promotes immune escape of hepatocellular carcinoma cells

**DOI:** 10.37349/etat.2023.00149

**Published:** 2023-06-30

**Authors:** Qiuyue Zhang, Jinchen Liu, Haifeng Lin, Bo Lin, Mingyue Zhu, Mengsen Li

**Affiliations:** Istituto Nazionale Tumori-IRCCS-Fondazione G. Pascale, Italy; ^1^Hainan Provincial Key Laboratory of Carcinogenesis and Intervention, Hainan Medical College, Haikou 571199, Hainan Province, China; ^2^Department of Medical Oncology, Second Affiliated Hospital, Hainan Medical College, Haikou 570216, Hainan Province, China; ^3^Institution of Tumor, Hainan Medical College, Haikou 570102, Hainan Province, China

**Keywords:** Hepatocellular carcinoma, reprogramming of glucose metabolism, immune escape

## Abstract

Hepatocellular carcinoma (HCC) is a complex process that plays an important role in its progression. Abnormal glucose metabolism in HCC cells can meet the nutrients required for the occurrence and development of liver cancer, better adapt to changes in the surrounding microenvironment, and escape the attack of the immune system on the tumor. There is a close relationship between reprogramming of glucose metabolism and immune escape. This article reviews the current status and progress of glucose metabolism reprogramming in promoting immune escape in liver cancer, aiming to provide new strategies for clinical immunotherapy of liver cancer.

## Introduction

Hepatocellular carcinoma (HCC) is one of the most common primary liver cancers, HCC is the sixth most common type of cancer worldwide. In recent decades, in many parts of the world, the occurrence of HCC and its related mortality has increased [1], and most patients have advanced beyond the transplant, surgery, or the standard of local treatment. In recent years, cancer immunotherapy has rapidly increased the number of drugs, which provide prognostic benefits by reactivating the immune system in response to tumors [2]. The microenvironment of cancer cells is completely different from that of normal cells. To adapt to changes in the nutritional status of the primary lesion, insufficient hemoperfusion, and hypoxic microenvironment, tumor cells must change their metabolic mode during the development of cancer, namely, metabolic reprogramming [3, 4]. Over the years, the energy metabolism of tumor cells has been known, while the concept and mechanism of metabolic reprogramming are now understood [5]. This is now considered a sign of tumor cells and promotes tumor growth [3], and glucose metabolic reprogramming plays an important part in the metabolic changes of tumors.

The interaction between tumors and the immune system is a continuous process. Immune editing can be divided into three stages: elimination, balance, and escape [6]. Tumor selection occurs in the second stage; when a subset of cancer cells persists, the immune response is sufficient to prevent cancer cell proliferation. When cancer cells that evade immune response become dominant, immune escape will happen [7]. Immune escape is a characteristic feature of malignant tumors. Tumor cells can survive and proliferate *in vivo* by avoiding recognition and attack by the body’s immune system. There is a mutual relationship between glucose metabolism and immune escape in liver cancer. During the reprogramming of glucose metabolism, cancer cells consume a large amount of glucose and produce lactic acid, which makes the tumor microenvironment (TME) in a state of low oxygen and energy. This has a great impact on the immune system and promotes cancer cells to escape immune surveillance. This article reviews the mechanisms of reprogramming glucose metabolism and immune escape in HCC cells.

## Overview of glucose metabolism reprogramming in cancer cells

### Glucose metabolism of normal cells

Glucose is the primary energy source for most mammalian cells. Glucose is first transported into cells through the glucose transporter (Glut) on the cell membrane and is then catabolized in the cell. The metabolic modes mainly include glycolysis, oxidative phosphorylation (OXPHOS), and the pentose phosphate pathway (PPP). Anaerobic oxidation of glucose is carried out in the cytoplasm, and the final step is phosphorylated at the substrate level by pyruvate kinase (PK), which generates two molecules of pyruvate [8]. Under normal oxygen conditions, pyruvate enters the mitochondria and decarboxylates to produce acetyl coenzyme A, which then circulates tricarboxylic acid (TAC) to release a large amount of energy. However, under hypoxic conditions, pyruvate is reduced to lactic acid by lactate dehydrogenase (LDH) in the cytoplasm, and 1 mol of glucose can produce only 2 mol of adenosine triphosphate (ATP). This metabolic mode is compensatory for normal cells in a hypoxic environment.

### Changes in glucose metabolism during the occurrence and development of liver cancer (glucose metabolism reprogramming)

**Figure 1:**
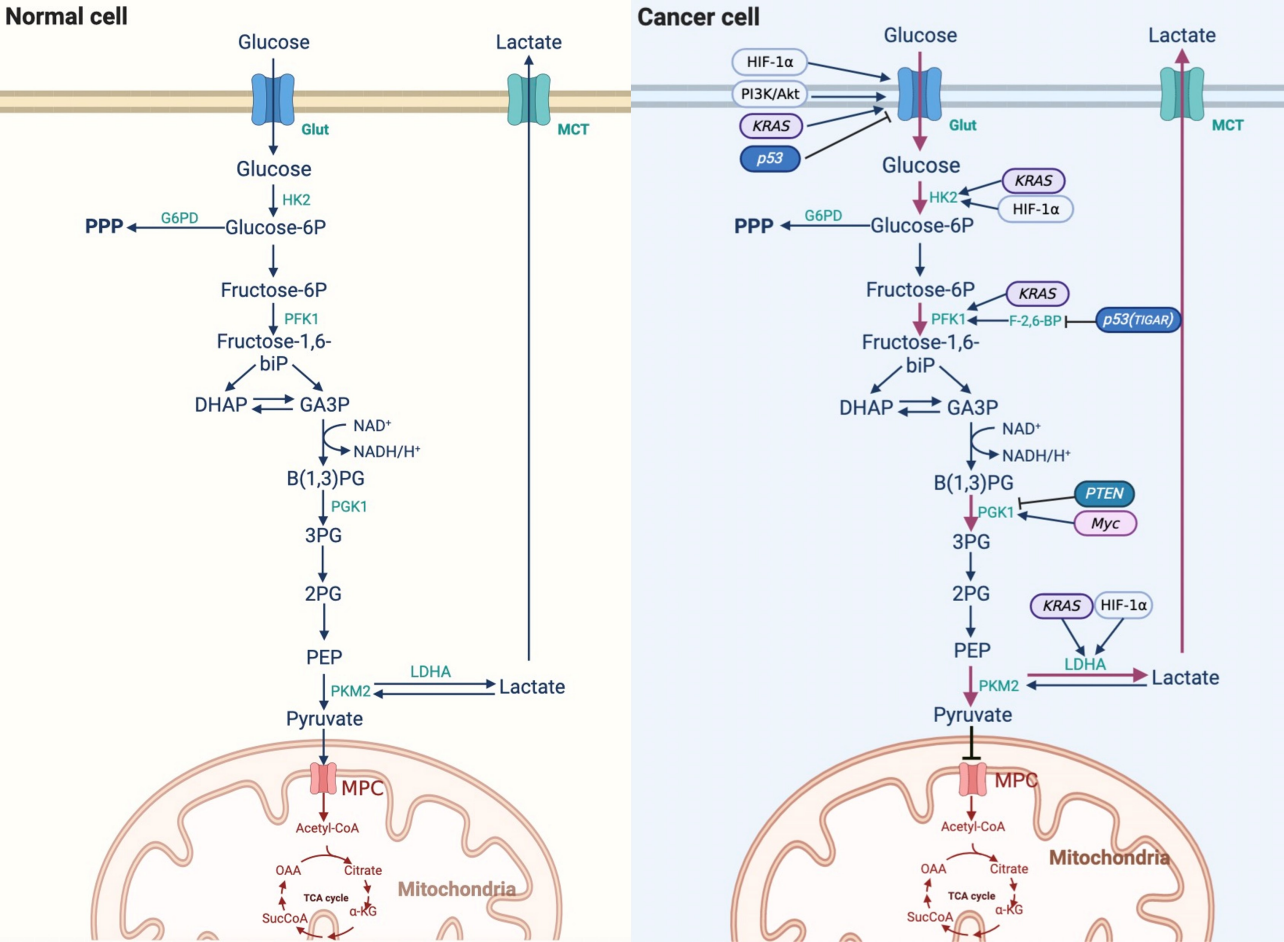
Difference of reprogramming of glucose metabolism between normal cells and cancer cells. 3PG: 3-phosphoglycerate; α-KG: α-ketoglutarate; Akt: protein kinase B; B(1,3)PG: 1,3-bisphosphoglycerate; DHAP: dihydroxyacetone phosphate; F-2,6-BP: fructose-2,6-diphosphate; GA3P: glyceraldehyde3-phosphate; HIF-1α: hypoxia-inducible factor-1α; HK2: hexokinase 2; KRAS: kirsten rat sarcoma viral oncogene; MCT: monocarboxylic acid transporter; MPC: mitochondria pyruvate carrier; NAD^+^: nicotinamide adenine dinucleotide (oxidation state); NADH: nicotinamide adenine dinucleotide (reducing state); OAA: oxaloacetic acid; PFK1: phosphofructokinase 1; PEP: phosphoenolpyruvate; PGK1: phosphoglycerate kinase 1; PI3K: phosphatidylinositol 3-kinase; PKM2: M2 isoform of pyruvate kinase; PTEN: phosphatase and tensin homolog; SucCoA: succinyl-coenzyme A; TCA cycle: tricarboxylic acid cycle

Glycolysis is necessary for cells to undergo oxidative glucose catabolism. In the 1920s, German physiologist Otto Warburg observed that tumor cells showed decreased oxygen consumption and increased glucose intake compared with normal cells. In addition, glucose ferments to produce lactic acid even in the presence of sufficient oxygen, indicating that tumor cells also tend to undergo glycolysis under normal oxygen conditions, hence the name aerobic glycolysis, or “Warburg effect” [9]. The transformation of tumor cells from anaerobic glycolysis to aerobic glycolysis is called reprogramming of glucose metabolism [10]. The Warburg effect shows that tumor cells rely on aerobic glycolysis to produce ATP compared with normal cells, which is dependent on OXPHOS [11, 12]. The results of ^18^F-deoxyglucose positron emission tomography (FDG-PET) showed that approximately 50–70% of ATP is produced by glycolysis in different tumors, and FDG-PET has also been used in the detection of liver cancer in recent years [13]. Numerous studies have shown that aerobic glycolysis is enhanced in liver cancer [14]. The PPP is a branch of glycolysis, which can generate nicotinamide adenine dinucleotide phosphate (NADPH) and phosphoribose, although it cannot produce ATP [11]. Glucose-6-phosphate dehydrogenase (G6PD), a key enzyme of the PPP, plays an important role in glucose metabolism. Hu et al. [15] showed that G6PD is involved in the invasion and migration of liver cancer cells, and immunohistochemistry showed that the expression of G6PD in liver cancer tissues was higher than that in the adjacent tissues. Compared to normal liver tissue, the expression of G6PD protein in liver tissue after hepatitis B virus (HBV) infection is higher. In liver cancer cells, the downregulation of G6PD induced by RNA interference reduces HBV replication five-fold through the interferon (IFN) pathway. Dore et al. [16] studied the clinical records of 11,143 patients and found that G6PD levels were positively correlated with liver cancer. These studies suggest that the expression of G6PD is increased and that the PPP is upregulated in HCC cells, which plays an important role in the development and formation of HCC. The differences in glucose metabolism between normal and cancer cells are shown in Figure 1.

### Activity changes of key enzymes related with glucose metabolism reprogramming

HK is the first rate-limiting enzyme in glycolysis, and four HK isoforms have been identified: HK1, HK2, HK3, and HK4 [17]. A fifth isoenzyme, containing hexokinase domain protein 1 (HKDC1), has recently been identified [18]. During the progression of HCC, the expression of HK changes from HK4 to HK2, leading to HCC cells that are insensitive to the anti-tumor activity of natural killer (NK) cells [18]. Lee et al. [19] showed that, compared with normal hepatocytes, the expression of genes involved in glycolysis and oxidative metabolism was higher in liver cancer tissues, and that HK2 may be a potential marker and molecular target for early detection and chemoprophylaxis of liver cancer.

PK is a key enzyme in glycolysis that catalyzes the phosphorylation of the substrate PEP to form pyruvate. PK has four isoforms: L isoform of pyruvate kinase (PKL), R isoform of pyruvate kinase (PKR), PKM1 and PKM2 [20]. PKM2 plays an important role in maintaining glucose metabolism in cancer cells. When PKM1 was used to replace PKM2, the metabolic mode of cancer cells changed from aerobic glycolysis to mitochondrial respiration [21]. PKM2 has both dimer and tetramer forms, and tetramers have a higher affinity for its substrate PEP and higher PK enzyme activity [20]. Li et al. [22] showed that PKM2 drives HCC progression by inducing an immunosuppressive microenvironment and upregulating programmed cell death ligand 1 (PD-L1) expression.

LDH reduces pyruvate conversion to lactate in the cytoplasm and is an important rate-limiting enzyme in the process of glucose metabolism. Active LDH is a tetramer synthesized by two different subunits, M and H, which are encoded by two genes, *LDHA* (M) and *LDHB* (H), respectively [23]. LDHA and LDHB have different abilities and directions for catalyzing the conversion of pyruvate and lactate. LDHA has a higher affinity for pyruvate and preferentially converts it to lactate under hypoxic conditions. However, LDHB has a high affinity for lactic acid, and converts lactic acid to pyruvate when oxygen is sufficient [24]. Sheng et al. [25] found that gene knockout of LDHA in the HCC cell line HCCLM3 by a lentiviral vector inhibited the growth and metastasis of cancer cells. These studies suggest that changes in the expression level and activity of LDHA in HCC cells promote the reprogramming of glucose metabolism in HCC cells and the malignant behavior of cancer cells.

## Influencing factors of glucose metabolism reprogramming in HCC cells

### HBV infection

Infection with the HBV is closely related to the occurrence and development of HCC. Although HBV does not directly promote the occurrence of HCC, numerous studies have shown that the hepatitis B virus X protein (HBx) plays an important role in the malignant transformation of human normal hepatocytes; however, its specific pathogenesis is still unknown [26]. HBx is a 154-amino acid regulatory protein with a molecular weight of approximately 17 kDa. It is one of the most conserved proteins in HBV subtypes, is mainly located in the cytoplasm, and plays an important regulatory role in viral replication and infection [27]. Niu et al. [28] found that HBx could up-regulate MicroRNA-155 in HCC cells, and microRNA-155 targeted the inhibition of PTEN expression and promoted malignant transformation of liver cancer. Studies have shown that HBV infection can affect glucose metabolism in host cells, the abundance of downstream metabolic intermediates of glucose is significantly increased, and HBx is an important upregulation factor of gluconeogenesis [29, 30]. HBV infection in hepatocytes may drive reprogramming of glucose metabolism.

HBV-infected hepatocytes preferentially express alpha-fetoprotein (AFP) and AFP receptor (AFPR). AFP can bind to PTEN and promote Akt phosphorylation, thereby activating the PI3K/Akt/mammalian target of rapamycin (mTOR) signaling pathway and promoting the malignant phenotype of hepatocytes. Activation of the PI3K/Akt signaling pathway is a key step in the HBx-driven malignant transformation of hepatocytes [31]. Li et al. [32] reported an interaction between AFP and PTEN. The binding of AFP to PTEN reduces the activity of PTEN, while the loss of the activity of PTEN and Akt is activated, which leads to the interruption of PTEN’s inhibitory signal on the PI3K/Akt pathway and causes the abnormal proliferation of HCC cells. The high-mobility group box transcription factor 1 (HBP1) has a tumor suppressor function. Cao et al. [33] showed that HBP1 inhibits AFP by binding to the affinity site of the AFP promoter. Inhibition of AFP by HBP1 attenuated the effect of AFP on the protein levels of PTEN, matrix metallopeptidase 9 (MMP9) and cysteine-dependent aspartate-specific proteases-3 (caspase-3) in HCC cells. HBx can inhibit the binding of HBP1 to the AFP promoter, leading to upregulation of AFP expression, which may promote tumorigenesis. Recent studies have shown that HBx can upregulate actin filament associated protein 1 like 2 (XB130), activate the PI3K/Akt pathway, and accelerate the progression of liver cancer [34]. It can be concluded that HBx upregulates AFP and promotes malignant growth of liver cancer cells through multiple pathways [35].

The PI3K/Akt/mTOR signaling pathway is abnormally activated in many tumorigenesis processes, participates in the regulation of cancer cell survival, promotes the proliferation, invasion, and metastasis of tumor cells [36], and can upregulate aerobic glycolysis in HCC. The PI3K/Akt pathway can regulate aerobic glycolysis in liver cancer through the following three mechanisms. First, the PI3K/Akt signaling pathway activates Glut1 and Glut4 to promote glucose transport into cells [37]. Second, PI3K/Akt signaling enhances the activity of glycolytic enzymes such as HK2 and PFK1 [38–40]. Third, PI3K regulates glucose metabolism via HIF-1α [41].

These findings suggest that HBV infection in human normal hepatocytes drives the expression of AFP, and the expression of AFP in the early stage of HCC can activate the PI3K/Akt signaling pathway, which is a key pathway for inducing glucose metabolism reprogramming.

### Glucose metabolism reprogramming is regulated by genes and proteins

*p53* plays a key role in inhibiting tumor progression, and recent studies have shown that mutant P53 protein drives metabolic reprogramming in cancer cells, indicating that *p53* plays an important role in regulating glucose metabolism. *p53* directly inhibits the transcriptional levels of Glut1 and Glut4 or indirectly inhibits the translocation of Glut1 to the cell membrane to reduce glucose uptake, and can also regulate glucose metabolism by regulating key enzymes in glucose metabolism [42]. Bensaad et al. [43] found a *p53*-inducing gene named *TIGAR*, whose expression can reduce the level of F-2,6-BP in cells, which is the strongest activator of PFK1, leading to glycolysis inhibition. *TIGAR* also reduces intracellular reactive oxygen species (ROS) through the PPP. The upregulation of mutant *p53* in HCC suggests that HCC cells may promote reprogramming of glucose metabolism due to changes in *p53* [44].

*PTEN* is a tumor suppressor gene that is often mutated or deleted in cancer and regulates glucose metabolism through the PI3K/Akt signaling pathway [45]. *PTEN* can reduce the rate of glycolysis and increase OXPHOS, thereby preventing the reprogramming of glucose metabolism in cancer cells [46]. PGK1 is a glycolytic enzyme whose activity is enhanced. Qian et al. [47] demonstrated that PTEN protein phosphatase regulates glycolysis by dephosphorylation and direct inhibition of PGK1’s tyrosine 324 (Y423) site. The inhibition of PGK1 autophosphorylation largely blocks glycolysis and brain tumor development, underscoring the important inhibitory role of PTEN protein phosphatase in regulating glycolysis in tumorigenesis. The high expression of AFP in HCC cells inhibited PTEN function, indicating that the reduction of PTEN function in HCC cells promoted the reprogramming of glucose metabolism.

*Myc* is the most common gene involved in carcinogenesis. *Myc* is composed of *c-Myc*, *N-Myc*, and *L-Myc* homologous genes. *Myc* can alter the TME and promote immune escape. In HCC models, it has been found that the translation phase of *Myc* is regulated by a specific protein, which is PD-L1. PD-L1 binds to programmed cell death 1 (PD-1) and promotes immune escape, which is important in Myc-mediated tumorigenesis [48]. Overexpression of *c-Myc* can lead to liver cancer in mice with enhanced glycolysis, and *c-Myc* is an important oncogene involved in the Warburg effect in liver cancer [14]. Xie et al. [49] showed that *Myc* could induce the expression of PGK1, improve the metabolic efficiency of liver cancer patients, and participate in the reprogramming of glucose metabolism in liver cancer cells.

*RAS*-mutated genes (including *HRAS*, *KRAS*, and *NRAS*) are among the most common oncogenes. Mutated *RAS* proteins stimulate downstream signals and have significant oncogenic effects, especially *KRAS* [50]. *RAS* family proto-oncogenes encode a group of small GTPases, whose activation can induce downstream signaling cascades, such as the mitogen-activated protein kinase (MAPK) pathway. Many studies have demonstrated that the RAS/MAPK pathway is associated with the development of human liver cancer [51, 52]. Treatment of HCC cells with specific mitogen-activated protein (MEK) inhibitors can lead to cell growth inhibition and apoptosis, indicating that the MAPK pathway plays a promoting role in the growth of HCC cells [52]. *KRAS* can upregulate Glut1 to increase glucose uptake, induce the expression of key glycolytic enzymes such as HK1, HK2, PFK1, and LDHA [51], and participate in the reprogramming of glucose metabolism in tumor cells. These findings indicate that tumor suppressor genes, oncogenes, and these genes expressed proteins are able to regulate the reprogramming of glucose metabolism in cancer cells.

### TME

Tumor tissues are composed of tumor cells, and the surrounding stroma undergoes significant changes during tumor formation [53]. This interaction between tumor cells and the surrounding environment forms the TME [54]. The TME includes three different cell components: endothelial cells, immune cells, and fibroblasts [55].

Tumor cells in the process of aerobic glycolysis produce a large amount of lactic acid, which is an important metabolic product in tumor metabolic reprogramming, the TME of pH value between 6.0 to 6.5, and stimulates fibroblast cells to produce hyaluronic acid, promoting the transfer of the tumor, invasion ability, increase of lactic acid, and immunosuppressive effects, leading to tumor escape immune surveillance, prolonging the survival time of tumors [56–58].

The hypoxic TME is a common characteristic of malignant tumors. A hypoxic TME can promote the transformation of tumor cells from OXPHOS to glycolysis, produce a large amount of lactic acid, and lead to cancer cell proliferation, immunosuppression, and resistance to immune attack of cancer cells [59]. HIF-1α is a major regulator of hypoxic stress in tumor cells. Activation of HIF-1α leads to the upregulation of Glut and glycolytic enzymes, especially the pyruvate dehydrogenase complex, HK and LDH [32]. The OXPHOS pathway in tumor cells is inhibited, while aerobic glycolysis is increased.

## Immune escape mechanism of HCC cells

### Immune cells and the immune escape of HCC cells

Cellular immunity is an important mechanism in immune editing. In the tumor environment, a large number of immune cells exist, including T lymphocytes, B lymphocytes, NK cells, dendritic cells (DCs), and macrophages, which play an anticancer role by inducing apoptosis and producing anti-tumor cytokines [60].

Tumor-infiltrating T cells, especially CD8^+^T cells, have been shown to play a critical role in tumor progression. Numerous studies have shown that T cell density is positively correlated with better prognosis in patients with HCC [61, 62]. Shigeta et al. [63] found that the combined treatment with regorafenib/PD-L1 normalized the blood vessels of liver cancer and increased the infiltration of CD8^+^T cells by increasing the expression of C-X-C motif chemokine 10 (CXCL10) in liver cancer cells, thus inhibiting tumor growth and improving the survival rate of liver cancer.

Regulatory T cells (Tregs) are a current research hotspot in the field of immunity. In the TME, Tregs are induced and differentiate from traditional T cells. Tregs have a powerful immunosuppressive effect, which can inhibit tumor immunity and promote the occurrence and development of tumors, and are key to inducing cancer cell immune escape [64]. Tregs can mediate immunosuppression in various ways [65]. Studies have shown that the number of Tregs in the peripheral blood of patients with liver cancer is higher than that in the blood of healthy people, and that Tregs in patients with advanced liver cancer are higher than those in patients with early liver cancer [66], indicating that Tregs are closely related to liver cancer progression and immune escape.

NK cells respond to target cells through a random combination of cell-surface activating and inhibitory receptors and have a broad spectrum of anticancer effects [67]. The NK cell surface-activating receptor binds to the ligand on the target cell surface to provide activation signals for NK cells to kill the target cells. Inhibitory receptors recognize type I major histocompatibility complex (MHC-I) on the surface of target cells and transmit inhibitory signals to tissue NK cells. NK cells account for 25–50% of the total number of liver lymphocytes, indicating that NK cells play an important role in liver immunity. Studies have shown that compared with healthy groups, the proportion of NK cells in the peripheral blood and liver tissues of HCC patients is reduced, which may help tumors evade immune surveillance [68].

Tumor-associated macrophages (TAMs) can be divided into two major groups, classically activated macrophages (M1) and alternatively activated macrophages (M2), which play different roles in the immune system and can be transformed into each other. M1 macrophages have anti-tumor effects, but M2 macrophages can promote the proliferation and invasion of tumor cells and can express a variety of cytokines to stimulate the proliferation and survival of tumor cells [69]. M2 macrophages and bone marrow-derived suppressor cells (MDSCs) inhibit the immune response of CD8^+^T cells and NK cells, promote HCC growth, and worsen the prognosis and survival of patients [70].

### Fas/Fas ligand signaling pathway and immune escape of HCC cells

Fas ligand (FasL) is a natural ligand of Fas that can specifically bind to Fas. After binding to Fas, FasL forms an active structure that can transmit signals to promote the formation of Fas trimers. The death domain in the Fas trimer binds to a death dome-related protein and transmits apoptotic signals to caspase-8. Caspase-8 is a protease, the activation of which induces apoptosis in Fas-expressing cells. Fas/FasL plays an important role in immune function, including induction of apoptosis and regulation of T cell activity, the interrelationship between the Fas/FasL signaling pathway and T cell receptor (TCR), and the expression of FasL, which controls the apoptotic and anti-apoptotic signaling pathways of T cells. The Fas/FasL signaling system also plays an important role in immune disorders of B cells [71].

The development of human tumors is often accompanied by the loss of Fas expression or function in tumor cells, but the expression of FasL is increased. Fas expression in liver cancer cells is significantly decreased, which reduces the binding of FasL to lymphocytes, resulting in the escape of lymphocytes. Fas is a common target of inactivation in tumorigenesis, and its induced apoptosis plays an important role in the biological effects of malignant tumors. In experimental animal models, the reduction of Fas can promote tumor development, whereas the recovery of Fas can delay the growth of primary tumors [72]. Studies have shown that single nucleotide polymorphisms of the Fas/FasL system are associated with the clinical and histopathological grades of HCC [73], and that the downregulated expression of Fas, upregulated expression of FasL in liver cancer cells, and elevation of soluble Fas in serum are important in the process of HCC cell evasion from immune surveillance [74].

### AFP and HCC cells immune escape

AFP plays an important role in HCC occurrence and development. AFP is produced during embryonic development and gradually decreases after the fetus is born, decreasing to a normal level at approximately 2 years of age and maintaining this low level. However, in the early stages of malignant transformation of hepatocytes, the *AFP* gene is highly expressed [75]. Currently, AFP is regarded as one of the main indicators for the diagnosis and prognosis of liver cancer in clinical practice. Studies have shown that when the concentration of AFP in serum is greater than 400 µg/L, the prognosis of patients with liver cancer is generally poor [76]. AFP can affect DCs and alter their anti-tumor effects. DCs are antigen-presenting cells (APCs) that can activate naive T cells and function in the uptake, processing, and presentation of antigens, while AFP can inhibit the maturation of DCs and induce their apoptosis [77]. After the treatment of DCs with AFP, the production of interleukin-12 (IL-12) and tumor necrosis factor-α (TNF-α) by DCs decreased. *In vivo* experiments showed that HCC patients with higher AFP levels had lower production of TNF-α by APC than healthy individuals [78]. Li et al. [79] found that AFP can block the caspase signaling pathway of tumor cells, activate the Fas/FasL interaction between HCC cells and lymphocytes, and promote the immune escape of liver cancer cells. These studies indicate that AFP plays an important role in the occurrence and development of HCC and participates in promoting the immune escape of HCC cells.

### Immune checkpoint and the immune escape of HCC cells

Cytotoxic T lymphocyte-associated antigen-4 (CTLA-4) and PD-1 immune checkpoints are negative regulators of T cell immune function, and the inhibition of these targets activates the immune system, leading to new immunotherapies for cancer [80]. After binding with its ligand B7, CTLA-4 generates inhibitory signals that inhibit T cell activation and protect tumor cells from T lymphocyte attack. The inhibition of CTLA-4 on T cells is mainly manifested by the competition between CTLA-4 and CD28. CTLA-4 and CD28 competitively bind to ligand B7. However, CTLA-4 has a higher affinity, and its function is contrary to that of CD28. After binding to B7, CTLA-4 generates inhibitory signals to block the effect of CD28 on T cells, thereby inhibiting their proliferation and activation of T cells [80, 81]. Overexpression of CTLA-4 in HCC leads to uncontrolled tumor growth [82], thus blocking the immune effect of CTLA-4 and stimulating the proliferation of immune cells, thereby inducing or enhancing anti-tumor immune responses. Anti-CTLA-4 monoclonal antibody can block the binding of CTLA-4 to its ligand and promote T cells to attack tumor cells, thus playing a role in anti-tumor activity. Agdashian et al. [83] found that treatment of HCC patients with tremelimumab, an anti-CTLA-4 drug, resulted in significant activation of T cell responses and detected corresponding biomarkers, which were helpful in identifying patients who responded to anti-CTLA-4 immunotherapy.

**Figure 2:**
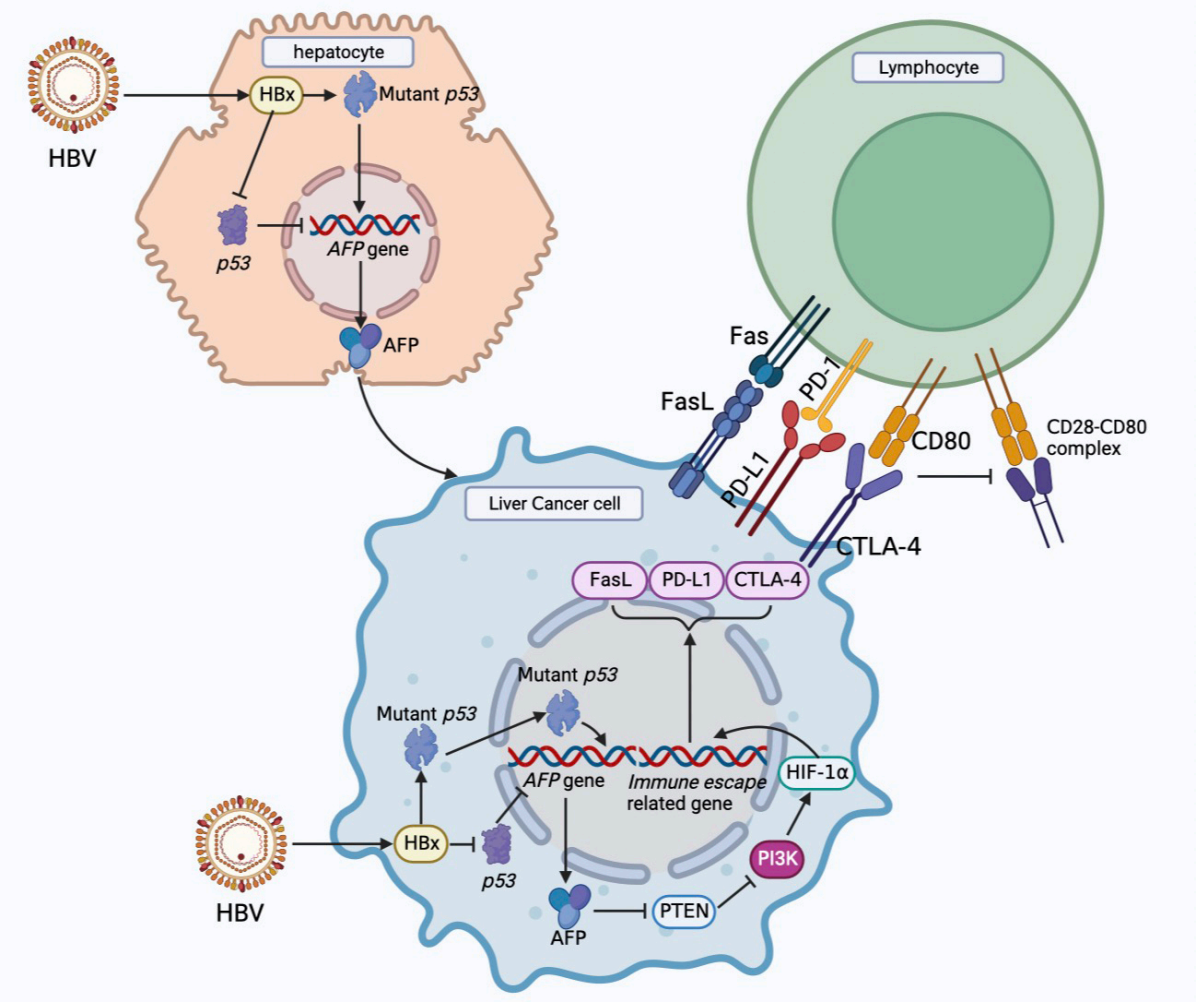
Schematic diagram of the mechanism of immune escape in HCC cells

PD-1 is mainly expressed in immune cells and plays an important role in suppressing immune responses and promoting self-tolerance by regulating T cell activity [84]. PD-L1 is highly expressed on the surface of tumor cells, and PD-L1 binding to PD-1 on the surface of T cells leads to T cell dysfunction and loss of antitumor ability. However, blocking the interaction between PD-1 and PD-L1 using anti-PD-1 or anti-PD-L1 antibody can reactivate T cells and release their antitumor activity [85]. In the HCC TME, PD-L1 is mainly expressed in Kupffer cells, with low expression in other APC or HCC cells. Kupffer cells with high PD-L1 expression interact with CD8^+^T cells expressing PD-1, leading to the dysfunction of effector T cells. In addition, increased expression of PD-L1 in HCC is positively correlated with poor prognosis in HCC patients and shortened overall survival [86]. PD-1/PD-L1 immune checkpoint inhibitors (ICIs) used for HCC monotherapy include nivolumab, pembrolizumab, and camrelizumab, which are mainly used as second-line therapies for HCC. Numerous studies have shown that HCC patients will benefit from PD-1/PD-L1 ICI treatment, and the combination of tyrosine kinase inhibitors (TKIs) and PD-1/PD-L1 ICIs will have better efficacy [82]. A schematic diagram of how AFP promotes immune escape of HCC cells and immunotherapies for HCC is shown in Figure 2.

## Reprogramming of glucose metabolism stimulates immune escape of HCC cells

### Effects of glucose metabolism reprogramming on immune cells

Clinical studies have shown that aerobic glycolytic activity in tumors cells is negatively correlated with the host anti-tumor immune response [87]. Glutamine in the human body can be converted from glucose, and glutamine inhibitors can suppress cancer cell oxidation and glycolysis, thereby reducing hypoxia and acidosis. Leone et al. [88] found that the use of glutamine inhibitors to block glutamine metabolism can inhibit tumor growth, not only reduce immune checkpoints, but also restore the antitumor function of immune cells. In conclusion, glucose metabolism in cancer cells can regulate the metabolism and function of the immune cells.

The high uptake of glucose in the TME may inhibit T cell function by affecting T cell metabolism. T lymphocytes use glucose and glutamine as their main energy sources. Glucose metabolism has a profound effect on the survival and function of T lymphocytes. Up-regulation expression of Glut1 and increased glucose uptake may stimulate T cell growth and cytokine secretion. However, overproduction of immune activation can lead to cancer or autoimmunity [89]. There is a metabolic interaction and nutrient competition between cancer cells and T cells, which may be key factors in carcinogenesis. The rapid growth of cancer cells and the increase in the Warburg effect consume nutrients in the surrounding microenvironment, and tumors deprive T cells of glucose, thus greatly reducing the effectiveness of the anti-tumor response of T cells [90, 91]. Evidences indicated that active glycolysis specifically facilitates the induction of proapoptotic FasL enhance TCR re-stimulation [92]. Moreover, there was a positive correlation between liver cancer glucose metabolism reprogramming and AFP high expression in cancer cells [93]. Due to AFP is able to inhibit the expression of Fas, it is speculated that liver cancer glucose metabolism reprogramming induces AFP expression and promotes liver cancer cells to evade immune surveillance.

The healthy liver is rich in a variety of cytokines and growth factors that stimulate hepatocyte growth; however, after liver tissue injury, Kupffer cells and liver-specific macrophages secrete proinflammatory cytokines. These pro-inflammatory factors play an important role in the early stages of tumor development [94]. Various properties of the TME can inhibit the function of NK cells, change their differentiation phenotype, prevent proliferation, and even induce NK cell apoptosis. In the TME, tumor-related cells secrete factors that prevent NK cell activation, including IL-6, IL-10, transforming growth factor-β (TGF-β), and prostaglandin E_2_ (PGE-2), to downregulate NK cell activation receptors, thereby inhibiting NK cell activation. Tumor-driven glucose restriction may reduce NK cell glycolysis and impair the antitumor function of NK cells [95].

The products of tumor glycolytic metabolism also have a negative impact on immune function. Lactic acid produced by tumor cells may upregulate the expression of IL-23 and IL-12 and can also strongly stimulate the IL-23/IL-17 proinflammatory pathway, and its mediated inflammation promotes tumorigenesis. The pathophysiological concentration of lactic acid also downregulates nuclear factor of activated T cells (NFATs) in T cells and NK cells, resulting in reduced production of IFN-γ. Clinical data show that the higher the tumor burden, the higher the serum concentration of lactic acid [87, 96, 97]. The pro-tumor effect of lactic acid may be related to its immunosuppressive effects. Lactic acid is a potent inhibitor of T and NK cell function and survival, leading to tumor immune escape.

Since lactic acid cannot cross the cell membrane by free diffusion, cells require a specific transport system provided by a proton-linked MCT to transport lactic acid along a concentration gradient. Fischer et al. [98] found that blocking MCT1 impaired T cell function, interfered with T cell metabolism, and inhibited T cell proliferation and cytokine generation by blocking lactic acid outflow from T cells. The accumulation of lactic acid and H^+^ and the low pH value in the TME can inhibit the activation of p38 and c-Jun N-terminal kinase (JNK)/c-Jun pathways triggered by TCRs to impair the function of CD8^+^T lymphocytes, thereby inhibiting their anti-tumor ability and causing tumor cell immune escape [99]. Lactic acid and low pH affect NK metabolism and function. Lactic acid directly inhibits NK cell toxicity by down-regulating the expression of NKp46 and interfering with the secretion pathway of NK cell-mediated cytotoxic activity. The reduction in toxicity is accompanied by low expression of perforin and granzyme in NK cells. Simultaneously, lactic acid can indirectly inhibit the toxicity of NK cells by increasing the number of MDSCs that inhibit NK cell toxicity [100].

Suppressor cells with immunosuppressive effects mainly include MDSCs, M2 macrophages, and Tregs. Husain et al. [100] injected lentivirus to interfere with pancreatic cancer cell Pan02 of LDHA in mice and found that the frequency of MDSCs in mouse spleen was decreased, while the activity of NK cells was higher, and exogenous lactic acid could increase the frequency of MDSC generation in mouse bone marrow cells cultured *in vitro*. An increase in lactic acid enhances immunosuppressive function and weakens innate immune function. M2-type macrophages secrete IL-6 and vascular endothelial growth factor (VEGF) to promote tumor metastasis and mediate tumor immune escape. Tumor-derived lactic acid can induce polarization of M2 macrophages by activating the extracellular regulated protein kinases (ERKs)/signal transducer and activator of transcription 3 (STAT3) signaling pathway and promoting angiogenesis and cancer cell migration [101].

**Figure 3:**
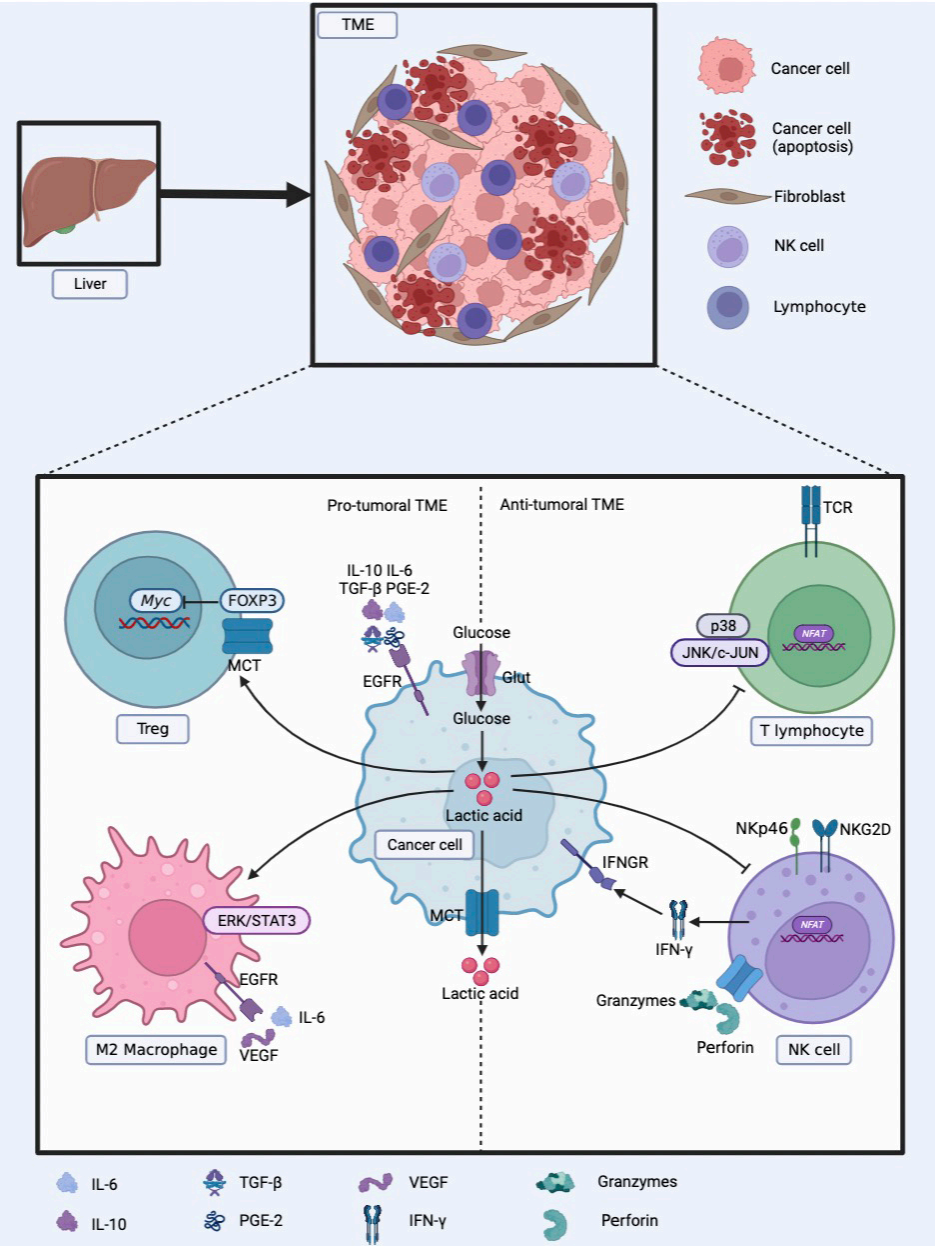
Reprogramming of glucose metabolism in HCC cells to escape surveillance of immune cells. EGFR: epidermal growth factor receptor; IFNGR: IFN-γ receptor; NKG2D: natural killer group 2, member D

In contrast, Treg cells are less dependent on glycolysis, their immunosuppressive effect depends on fatty acid metabolism and OXPHOS, and they are more tolerant to high lactic acid environments. The lactic acid-rich microenvironment enhances the viability of Tregs and their immunosuppressive abilities. Lactic acid can weaken the proliferation of T cells, but the proliferation and function of Tregs are not negatively affected. Forkhead box protein 3 (FOXP3) can inhibit the transcription of Myc, glycolysis, and make Tregs resistant to lactic acid inhibition. FOXP3 can affect the response of LDH by oxidizing lactic acid to pyruvate. This protective mechanism enables Tregs to tolerate acidic microenvironment [102]. Tregs infiltrated by tumors can take up lactic acid to maintain their immunosuppressive function by increasing the expression of FOXP3 and MCT1 [103]. Reprogramming of glucose metabolism in HCC cells can stimulate the escape surveillance of immune cells, as shown in Figure 3.

### Effects of glucose metabolism reprogramming on immune checkpoints

PKM2, a key enzyme in the process of glucose metabolism reprogramming, can catalyze the conversion of the substrate PEP to pyruvate, and PKM2 has two different spatial conformations, dimers and tetramer, which can be interconverted. Tetramer PKM2 has a high PK enzyme activity, whereas PKM2 has a low activity. This is responsible for the regulation of glycolysis. Dimer PKM2 acts as a coactivator of HIF-1α, and HIF-1α binds directly to the DNA hypoxia transcript site of PD-L1, resulting in increased PD-L1 expression [104]. Guo et al. [105] and Long et al. [106] found that the expression of PKM2 and PD-L1 was positively correlated in lung adenocarcinoma patients, the prognosis of patients with high expression of PKM2 and PD-L1 in tumor cells and immune cells was poor, and the PD-L1 level in lung adenocarcinoma cells was significantly downregulated after PKM2 knockdown. Increasing the tetramer ratio of PKM2 or silencing PKM2 mRNA can downregulate PD-L1 and reduces immune escape.

IFN-γ secreted by T cells induces tumor cells to express PD-L1. The PD-1/PD-L1 interaction inhibits T cell function and enables tumors to escape immune attack. After treatment of tumors with PD-L1 monoclonal antibody, the expression of glycolytic enzymes and the phosphorylation level of Akt decreased, which inhibited glycolysis, and the expression of PD-L1 in tumor cells was positively correlated with the rate of glycolysis. The expression of PD-L1 in tumor cells drives the activation of the PI3K/Akt signaling pathway and stimulates aerobic glycolysis. Enhanced glucose uptake by cancer cells and enhanced competition for glucose by T cells. After the use of a PD-L1 blocker, more glucose can remain in the TME [90, 107]. These results indicate that reprogramming of glucose metabolism influences the expression of immune checkpoints and stimulates the immune escape of HCC cells.

## Outlook

Compared with normal cells, the characteristics of cancer cells preferentially use glycolysis to provide energy, so focusing Glut and glycolytic enzymes may become cancer treatment targets, such as targeting Glut, inhibiting activity of HK2 and PFK1, and using MCT1 and MCT4 inhibitors to selectively target the glucose metabolism reprogramming in cancer cells. Recently, regulating metabolic reprogramming has become a new therapeutical target for HCC [108, 109]. As mentioned above, the large amount of lactic acid produced by the glycolysis of cancer cells will damage the function of T cells, and the microenvironment of glucose depletion will inhibit the activity of T cells, thus enabling cancer cells to escape the immune surveillance of T cells. Another mechanism of immune escape is achieved by the combination of PD-1 receptor on the surface of T cells and PD-L1 ligand on the surface of tumor cells. Evidence indicated that IFN-α remodels glucose metabolism in TME to potentiate anti-PD-1 efficacy in treating HCC [13]. Therefore, the combination of targeted glucose metabolism and immunotherapy may produce synergy and enhance the effect of immunotherapy.

Reprogramming glucose metabolism in HCC cells is an important natural regulation mechanism for cancer cells to survive in hypoxic environments. Reprogramming of glucose metabolism can promote the immune escape of HCC cells, which brings great constraints to HCC immunotherapy. Exploring the reprogramming of glucose metabolism in HCC cells can reveal how to initiate the reprogramming program of glucose metabolism in the early stage of HCC and identify the source of the immune escape of HCC cells. It is a key strategy that effectively prevents the occurrence and development of liver cancer, inhibits glucose metabolic reprogramming, and increases the sensitivity of cancer cells to immune therapy. An in-depth study of glucose metabolic reprogramming, which may reveal the regulatory mechanism of glucose metabolic reprogramming involved in escaping immune surveillance of HCC cells, will provide new strategies for HCC immunotherapy.

## Abbreviation

AFP: alpha-fetoprotein
Akt: protein kinase B
APCs: antigen-presenting cells
ATP: adenosine triphosphate
caspase-3: cysteine-dependent aspartate-specific proteases-3
CTLA-4: cytotoxic T lymphocyte-associated antigen-4
DCs: dendritic cells
FasL: Fas ligand
FOXP3: forkhead box protein 3
G6PD: glucose-6-phosphate dehydrogenase
Glut: glucose transporter
HBP1: high-mobility group box transcription factor1
HBV: hepatitis B virus
HBx: hepatitis B virus X protein
HCC: hepatocellular carcinoma
HIF-1α: hypoxia-inducible factor-1α
HK2: hexokinase 2
ICIs: immune checkpoint inhibitors
IFN: interferon
IL-12: interleukin-12
KRAS: kirsten rat sarcoma viral oncogene
LDH: lactate dehydrogenase
MAPK: mitogen-activated protein kinase
MCT: monocarboxylic acid transporter
MDSCs: marrow-derived suppressor cells
NFATs: nuclear factor of activated T cells
NK: natural killer
OXPHOS: oxidative phosphorylation
PD-1: programmed cell death 1
PD-L1: programmed cell death ligand 1
PEP: phosphoenolpyruvate
PFK1: phosphofructokinase 1
PGE-2: prostaglandin E_2_
PGK1: phosphoglycerate kinase 1
PI3K: phosphatidylinositol 3-kinase
PK: pyruvate kinase
PKM2: M2 isoform of pyruvate kinase
PPP: pentose phosphate pathway
PTEN: phosphatase and tensin homolog
TCR: T cell receptor
TGF-β: transforming growth factor-β
TME: tumor microenvironment
Tregs: regulatory T cells
VEGF: vascular endothelial growth factor

## Declarations

### Availability of data and materials

All data generated or analyzed during this study are included in this manuscript.

### Copyright

© The Author(s) 2023.
